# Modulation of neuroinflammation and oxidative stress by targeting GPR55 – new approaches in the treatment of psychiatric disorders

**DOI:** 10.1038/s41380-024-02614-5

**Published:** 2024-05-25

**Authors:** Matthias Apweiler, Soraya Wilke Saliba, Lu Sun, Jana Streyczek, Claus Normann, Sabine Hellwig, Stefan Bräse, Bernd L. Fiebich

**Affiliations:** 1 Neuroimmunology and Neurochemistry Research Group, Department of Psychiatry and Psychotherapy, Medical Center - University of Freiburg, Faculty of Medicine, University of Freiburg, D-79104 Freiburg, Germany; 2 Department of Cardiology and Angiology, Medical Center - University of Freiburg, Faculty of Medicine, University of Freiburg, D-79106 Freiburg, Germany; 3 Department of Psychiatry and Psychotherapy, Medical Center - University of Freiburg, Faculty of Medicine, University of Freiburg, D-79104 Freiburg, Germany; 4 Institute of Organic Chemistry, Karlsruhe Institute of Technology (KIT), Kaiserstrasse 12, D-76131 Karlsruhe, Germany; 5 Institute of Biological and Chemical Systems-Functional Molecular Systems (IBCS-FMS), Karlsruhe Institute of Technology (KIT), Kaiserstrasse 12, D-76131 Karlsruhe, Germany

**Keywords:** Psychiatric disorders, Drug discovery

## Abstract

Pharmacological treatment of psychiatric disorders remains challenging in clinical, pharmacological, and scientific practice. Even if many different substances are established for treating different psychiatric conditions, subgroups of patients show only small or no response to the treatment. The neuroinflammatory hypothesis of the genesis of psychiatric disorders might explain underlying mechanisms in these non-responders. For that reason, recent research focus on neuroinflammatory processes and oxidative stress as possible causes of psychiatric disorders. G-protein coupled receptors (GPCRs) form the biggest superfamily of membrane-bound receptors and are already well known as pharmacological targets in various diseases. The G-protein coupled receptor 55 (GPR55), a receptor considered part of the endocannabinoid system, reveals promising modulation of neuroinflammatory and oxidative processes. Different agonists and antagonists reduce pro-inflammatory cytokine release, enhance the synthesis of anti-inflammatory mediators, and protect cells from oxidative damage. For this reason, GPR55 ligands might be promising compounds in treating subgroups of patients suffering from psychiatric disorders related to neuroinflammation or oxidative stress. New approaches in drug design might lead to new compounds targeting different pathomechanisms of those disorders in just one molecule.

## Introduction

A meta-analysis, including data from 27 countries all over the world and covering findings from 1985 to 2012, showed that the prevalence of any psychiatric disorder was 13.4% in children and adults [1]. Another meta-analysis covering the period 1980 to 2013 and 59 countries even estimated a period prevalence of 17.6% for psychiatric disorders and a lifetime prevalence of 29.2% [2]. Therefore, more than one out of four will be affected by a psychiatric disorder over his lifespan, underlining the importance of adequate mental health care and effective treatment. In general, epidemiological evidence shows that the incidence and prevalence of psychiatric disorders are not rising, but due to reduced stigma, more psychiatric disorders will be diagnosed, leading to a formal increase over the last decades [1].

According to the Global Burden of Disease Study 2019, psychiatric disorders lead to 14.6% of all years lived with disability and increase the all-cause mortality by suicide, reduced help-seeking behavior, and less physical health treatment [3]. The rising number of diagnosed psychiatric disorders with high life prevalence and the medical and social consequences of these disorders highlights the need for high-effective therapy.

The etiology and the molecular pathological mechanisms of psychiatric and neuropsychiatric diseases, such as Alzheimer’s Disease (AD), are still the subject of studies and remain poorly understood. Based on the positive effects of the currently used pharmacological treatments, such as tricyclic antidepressants (TCAs), selective serotonin reuptake inhibitors (SSRIs), and antipsychotics, monoamines and their receptors in the CNS are considered an important part of the pathogenesis of psychiatric disorders [4]. This led, together with reduced monoamine serum levels, to the monoamine deficiency hypothesis [5, 6], which is controversial nowadays since it cannot sufficiently explain the genesis of psychiatric disorders [7]. Furthermore, the present pharmacological treatment based on the modulation of the monoamine systems is not relieving the psychiatric symptoms in every patient. In depression, only a third of the patients show an adequate response or full remission of their symptoms in the first 8 weeks of psychopharmacotherapy [8], emphasizing the need for further research on the pathomechanisms of psychiatric disorders. Inflammatory processes are gaining more attention in recent molecular psychiatric research. Increased levels of pro-inflammatory cytokines are found in patients suffering from different psychiatric and neuropsychiatric disorders [9], with anti-inflammatory treatment (celecoxib or acetylsalicylic acid) showing significant effects on relieving the symptoms in first trials [10]. The search for biomarkers predicting the success of either monoamine reuptake therapies or anti-inflammatory treatment would represent an important step to individualization in pharmacotherapy and may lead to etiological-biological subclassification of psychiatric disorders, such as depression or schizophrenia.

With an increased understanding of possible molecular mechanisms involved in the pathophysiology of psychiatric and neuropsychiatric disorders, the development of specific drugs targeting the underlying processes gains importance. G-protein coupled receptors (GPCRs) have been identified as a large and important class of receptors in the pathogenesis of psychiatric disorders. Most neurotransmitters considered relevant to psychiatric disorders are enfolding their physiological effects via different types of GPCRs [11]. Therefore, new therapeutical approaches in treating psychiatric disorders should focus on GPCRs and ways to modulate inflammatory and other driving pathological conditions in the CNS resulting in the disorders’ symptoms.

This non-systematic literature review investigates the role of neuroinflammation and oxidative stress in the genesis of psychiatric disorders. Furthermore, the role of the G-protein coupled receptor 55 (GPR55) in inflammatory and oxidative pathomechanisms and, therefore, treatment of psychiatric disorders will be discussed. Finally, the coumarin scaffold will be considered as a promising natural structure for pharmacological drug design. Literature search was conducted using the PubMed® database and the search engine Google scholar. Keywords such as “GPR55”, “neuroinflammation”, “oxidative stress” and the different agonists, antagonists, and psychiatric disorders were chosen. Based on the found literature, cross-references were additionally used.

## Inflammation and oxidative stress contribute to psychiatric disorders

Inflammatory processes are considered to be associated with psychiatric disorders. Especially chronic low-grade neuroinflammation, a slight disbalance towards pro-inflammatory mediators, is understood as a driving factor of psychiatric disorders [12]. A retrospective study covering over 12,000 patients suffering from immune-mediated inflammatory diseases (IMID), such as inflammatory bowel diseases (IBD) or multiple sclerosis (MS), showed a higher incidence of psychiatric disorders if matched to healthy controls [13]. A systematic review focusing on psychiatric disorders in MS patients demonstrated higher rates of depression and anxiety disorders in MS patients [14]. Higher incidences of major depression were found in patients with an IBD, especially in females, with aggressive and active disease serving as strong predictive factors [15]. Severe infections and autoimmune diseases are associated with a higher risk for schizophrenia spectrum disorders, with the highest risk for patients suffering from autoimmune diseases with CNS-specific antibodies and severe infections in their previous history [16]. The advances in the understanding, diagnostics, and treatment of autoimmune encephalitis and autoimmune-antibody-associated neuropsychiatric syndromes resulting in psychotic symptoms clearly support the immunological and inflammatory pathomechanistic theories for at least a subgroup of patients [17].

A Swedish population- and a sibling-based study reported higher incidences of autoimmune diseases after exposure to traumatic or stressful events with the development of a stress-related disorder, such as PTSD. Interestingly, using SSRIs during the first year after the diagnosis of PTSD showed decreased incidences of autoimmune diseases [18]. For major depression, a decrease of interleukin (IL)-6 serum levels based on the treatment with SSRIs has been shown before [19], eliciting possible anti-inflammatory mechanisms of SSRIs besides the increase of serotonin. Besides pharmacological treatment, cognitive behavioral therapy did not only decrease depressive symptoms in unmedicated first-episode women, but also reduced IL-6 baseline levels. Even if this study did not show a correlation between symptoms’ severity and the IL-6 baseline levels, remission rate and variation of the IL-6 levels were correlated [20]. Conversely, high IL-6 baseline levels in patients with depression were negatively correlated with the response to SSRIs or serotonin-norepinephrine reuptake inhibitors (SNRIs) [21], supporting the hypothesis of different subgroups with different pathomechanisms in the genesis of depressive disorders. This study suggests high IL-6 levels as a possible predictor of SSRI-/SNRI failure and shows a positive correlation between IL-6 levels and the severity of depressive symptoms [21]. Furthermore, higher serum levels of tumor necrosis factor (TNF)α as well as lower levels of brain-derived nuclear factor (BDNF), were found in depressive patients, with an association between BDNF levels and the severity of the depression [21].

Besides the association of autoimmune diseases and psychiatric disorders, altered levels of pro- and anti-inflammatory mediators have been described in the serum and cerebrospinal fluid of patients suffering from psychiatric disorders [9]. Besides raised levels of inflammatory cytokines and mediators, the C-reactive protein (CRP), commonly used as inflammatory marker, is increased in different psychiatric disorders, such as depression and schizophrenia [22]. In bipolar disorder, raised cytokines, such as IL-1β, microglial activation, and an alteration of arachidonic acid cascade were shown in post-mortem brain analyses [23, 24]. In elderly patients, increased serum levels of IL-1β and IL-6 in depression [25] and increased levels of IL-1β [25], as well as microglial activation markers [26] in AD, are reported.

The CNS is the main utilizer of oxygen and has a great need for energy. Therefore, the CNS is highly exposed to reactive oxygen species (ROS) inducing oxidative stress, especially if cellular anti-oxidative mechanisms fail [27]. Changes in energy metabolism and oxidative damage have been found in depressive patients compared to age-matched controls, suggesting a connection between oxidative stress and depression [28]. A reduced gene expression of anti-oxidative enzymes has been described in oligodendrocytes of the Locus coeruleus and the occipital cortex in post-mortem brain analyses of depression compared to healthy controls [29]. In schizophrenia, oxidative stress, measured by an increase of oxidized proteins compared to the controls, is considered an important factor in the onset and progress of the disease, besides other factors [30]. Furthermore, genetic alterations in oxidative pathways are associated with schizophrenia [31]. An MR-spectroscopy study measuring intracortical glutathione levels in patients with schizophrenia reported a positive association between higher intracortical glutathione levels and better outcomes in first-episode schizophrenia [32].

Aβ-aggregates in AD seem to be connected to increased oxidative stress, measured by increased lipid peroxidation products, causing neurodegeneration and the progress of the disease [33]. In AD mice models, anti-oxidative treatment reversed neurodegeneration in Nissl staining of the hippocampus and cortex [34], supporting the key role of oxidative stress in AD.

G-protein coupled receptors form a receptor superfamily with over 800 identified receptors [35]. About 33% of the known pharmaceutics target members of the GPCR family, mediating their effects via GPCRs and their associated pathways [36]. Different signaling proteins, such as the trimeric G-Protein complex (Gαβγ) or β-arrestins, are bound to the receptor and are responsible for the different intracellular effects after receptor activation [37].

The importance of GPCRs and their possible role in the pathomechanism of psychiatric diseases is supported by the effects of pharmacological treatment of those diseases. Most of the receptors directly or indirectly targeted by antidepressants, anxiolytics, and antipsychotics belong to the GPCR family, and the effects of the drugs on the receptors are associated with reduced symptoms in psychiatric diseases [38].

Since GPCRs modulate inflammatory and oxidative cellular pathways, they are interesting pharmacological targets to interfere with the interconnection of inflammation and oxidative stress in pathological conditions, such as psychiatric disorders. GPR55 has gained attention in the last decades, and various signal pathways associated with inflammation and oxidative stress are controlled by this receptor. The pathways modulated by GPR55 might contribute to psychiatric and neuropsychiatric pathologies, therefore, targeting this receptor might enfold beneficous effects in some disorders [39–43].

## GPR55 in neuroinflammation, oxidative stress, and psychiatric disorders

GPR55 is a deorphanized GPCR [44] initially identified as a 981 base pair and 319 amino acid long gene/protein in humans [45]. GPR55 is widely distributed in peripheral and central nervous tissues, with the highest expression in the frontal cortex, striatum, hippocampus, hypothalamus, and other regions of the CNS expressed on glial cells and neurons [46]. Although GPR55 only shares a sequence homology of 13.5% to the cannabinoid receptor (CB) 1 and 14.4% to the CB2 [44], GPR55 is discussed as a non-CB1, non-CB2 cannabinoid GPCR but modulator of the endocannabinoid system [47]. Numerous agonists and antagonists targeting GPR55 have been identified and designed that can be divided into endogenous and synthetic ligands and full, partial, or inverse agonists/antagonists at this receptor [44]. Phytocannabinoids such as delta-9 tetrahydrocannabinol (Δ^9^THC) or synthetic cannabinoids such as O-1602 act as GPR55 agonists, while the phytocannabinoid cannabidiol (CBD) or the synthetic ML 193 act as antagonists [48]. Endogenous lipid mediators such as lysophosphatidylinositol (LPI) or anandamide are discussed as another class of GPR55 agonists [48]. LPI has been described in numerous pathologies [49–53]. As an endogenous GPR55 agonist, the immunogenicity and digestibility might be better as compared to synthetic receptor agonists and antagonists. However, as an orthosteric GPR55 agonist, undesired effects in different tissues can be triggered; high LPI concentrations are associated with metabolic disorders, such as obesity and diabetes and can booster cell proliferation and therefore neoplasia [49, 52, 53]. Therefore, allosteric receptor ligands might offer a more selective and distinct modulation of different receptor states with less side effects [54]. LPI is suggested to be the main endogenous ligand of GPR55 with an EC_50_ of 200 nM for the phosphorylation of extracellular signal-regulated kinases (Erk) in GPR55-HEK293 cells since the observed effects on the Erk-phosphorylation were GPR55 dependent and outshined other tested compounds [55]. Furthermore, LPI induces intracellular calcium release and increased binding of [^35^S]GTPγS to GPR55-HEK293 cell membranes [55], suggesting activation of GPR55-associated G-proteins.

**Fig. 1:**
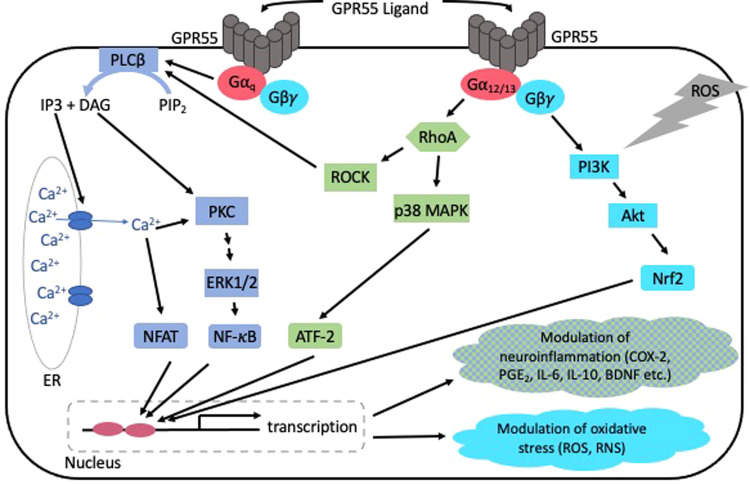
Simplified overview of GPR55 dependent signaling pathways and resulting effects [50, 58, 60, 71, 85, 93].

For GPR55, Gα_12,_ Gα_q_ and Gα_13_ [56] dependent signaling have been reported, leading to the activation of phospholipase (PLC) and GPR55-dependent RhoA [57]. Gα proteins coupled to GPR55 kick off further downstream signal pathways, such as the mitogen-activated protein kinases (MAPK)-pathway with phosphorylation of Erk1/2, the activation of transcription factors [50] such as Nrf2 [58], and in consequence changed gene expression and protein synthesis (Fig. 1). However, another intracellular signaling can be activated by the known GPR55 agonists [59], and antagonists with inverse agonistic properties [60].

The role of GPR55 in psychiatric disorders is still under investigation, but based on the signal pathways, GPR55 is associated with inflammatory responses modulated via this receptor. Functional polymorphisms of GPR55 led to altered Erk1/2 phosphorylation in Chinese hamster ovary (CHO) cells, and GPR55 Val195 polymorphism was associated with increased vulnerability to anorexia nervosa in Japanese females [61]. In the dorsolateral prefrontal cortex of suicide victims, post-mortem analysis showed reduced GPR55 mRNA and protein levels but an increased occurrence of GPR55-CB2 heterodimers, identified in neurons and astrocytes [43]. Additionally, a dysregulation of the PI3K/Akt/GSK3β pathway contributing to autism spectrum disorder (ASD) due to its importance for differentiation and survival of nervous cells has been shown. GPR55 modulation by prenatal treatment with LPI in a valproic acid induced murine autism-model prevented neuronal and synaptic abnormalities by an increase of phospho-Akt and phospho-GSK3β expression [62]. Furthermore, GPR55 activation by O-1602 might be beneficial in nicotine use disorder via the PI3K/Akt pathway due to an inhibition of nicotine-induced Akt phosphorylation [63]. Activation of GPR55 by O-1602 enhanced glutamate but not dopamine release in the nucleus accumbens, associated with a reduced intravenous nicotine self-administration of rats and mice. Effects of O-1602 on nicotine self-administration were repealed in GPR55 knockout mice and cocaine self-administration was not altered by GPR55 activation, suggesting the effects to be limited to nicotine use disorders [64].

### Modulation of neuroinflammation via GPR55

Neuroinflammation is considered a disbalance towards pro-inflammatory mediators in central nervous tissues as a reaction to an injury, intoxication, or as a cause or reaction to diseases [65] that potentially harms the physiological functions of the CNS. Glial cells are the main initiators and regulators of inflammatory processes in the CNS, which not only affect themselves but damage neurons and lead to neurodegeneration [66]. Damage- or pathogen-associated molecular patterns (DAMPs/PAMPs), such as extracellular ATP, uric acid, lipopolysaccharide (LPS), or Flagellin, act as initiators for neuroinflammatory processes via receptors on microglial cells and astrocytes [12]. Important neuroinflammatory mediators are cytokines, such as IL-1β, IL-6, and TNFα, chemokines, and second messengers, such as prostaglandins (PG) and nitric oxide (NO) [67]. Those mediators are normally balanced with anti-inflammatory mediators, such as soluble IL-1 receptors, IL-10, brain BDNF, and nerve growth factor (NGF) [66].

An emerging body of research underlines the role of GPR55 in regulating neuroinflammatory processes in the CNS. Activation of GPR55 using O-1602 ameliorated IL-1β-induced reduction of neuronal formation and increased neurogenesis in neuronal stem cells in vitro [68]. In vivo, however, LPS induced chronic inflammation and activation of microglia, O-1602 neither affected microglial activation in wild-type C57BL/6 nor in GPR55 knockout mice [68]. In the microglial BV-2 cell line, administration of LPI reduced LPS-induced IL-6 and TNFα synthesis. Neither LPS nor LPI stimulation induced or affected the synthesis of IL-1β, IL-2, IL-10, IL-12, and interferon (IFN)γ [69]. In C57BL/6J mice, chronic social defeat stress (CSDS) induced depression- and anxiety-like behavior that was associated with a reduced GPR55 protein synthesis with maintained GPR55-mRNA expression in hippocampal areas of susceptible mice. Activation of GPR55 using O-1602 inhibited the activation of the inflammasome, normalizing the increased expression of IL-1β (IL-6 and TNF) and the decreased expression of IL-4 and IL-10, respectively reducing the depression- and anxiety-like behavior [70]. Another study focusing on anxiety-like behavior of male Wistar rats using the elevated plus-maze test, showed anxiolytic effects of intracerebroventricular O-1602 injections, while the commercial GPR55 antagonist ML 193 increased anxiety-like behavior [42]. GPR55-deficient mice showed increased pro-inflammatory cytokines IL-1β, IL-6, and TNFα in conditions of chronic low-grade inflammation when compared to wild-type mice [68]. The presented results support an anti-inflammatory function of agonistic GPR55 receptor activation.

Contrasting the previous results, the commercial GPR55 antagonist ML 193 but not the agonist O-1602 reduced LPS-induced PGE_2_-release in primary rat microglia [71]. CID16020046, a commercial GPR18/GPR55 antagonist, showed a reduction of LPS-induced IL-6 and COX-2 expression in primary microglial cultures of Wistar rats [72]. These anti-inflammatory effects might be explained by biased agonistic activities of those antagonistic compounds at GPR55.

The basic principles of agonism and antagonism at GPCRs need to be explained to understand inverse or biased agonism. GPCRs, such as GPR55, show basal activity even without being bound by a ligand through spontaneous changes between active and inactive states of the receptors [73]. Agonists shift the likelihood to receptor activation, while classical antagonists reduce the likelihood of receptor activation, preventing agonists from binding (competitive or allosteric inhibition) and maintaining or even reducing the basal receptor activity by stabilizing the inactive receptor conformation [73]. However, some ligands can even reduce the constitutive activity of GPCRs and are referred to as inverse agonists [74]. Depending on the efficacy of the induced change of activity in either direction, partial and full (inverse) agonists and antagonists can be distinguished [73]. More recently, it has been shown that various receptor states might lead to a differentiated activation of G-Proteins or β-arrestins and variable functionality of the activation of those transducers, explaining the concept of biased agonism [75]. Therefore, different agonists might enfold distinct effects via the same receptor depending on their chemical structure, interaction with the receptor, and the differentiated activation of the transducer. Different ligands may stabilize individual active states of GPCRs, connected to distinct and sometimes even contrasting intracellular pathways and, therefore cellular responses [76].

Biased agonism leads to new ways of understanding the unexpected actions of known antagonists at GPR55. LPI and virodhamine, another GPR55 agonist, induced intracellular calcium release via GPR55 in GPR55-transfected HEK293 cells. However, LPI mainly acted via Gα_q_ and PLCβ, while the effects of virodhamine were dependent on Gα_13_, PLCε, and the Rho-/ROCK-pathway, demonstrating biased agonism at GPR55 [59]. In intestinal inflammation, protection against inflammatory processes due to treatment with the GPR55 agonist O-1602 [77] and the GPR55 antagonist CID16020046 [78] was observed, further supporting biased agonism at GPR55. We have shown inverse agonistic activities leading to anti-neuroinflammatory cellular responses, such as reduction of PGE_2_ and IL-6, for numerous coumarin-derivates with a high antagonistic affinity for GPR55 [60, 71, 79]. In line with biased agonism, the observed effects of the coumarin-derivates varied depending on the specific chemical modifications introduced to the coumarin scaffold [60].

In psychiatric and neuropsychiatric disorders, the role of GPR55 remains the focus of ongoing research. It has been shown that O-1602 ameliorated chronic social defeat stress-induced depression- and anxiety-like behavior in mice by modulating inflammatory processes [70]. Furthermore, O-1602 attenuated the depression-like behavior of female Wistar rats [41]. Shi et al. reported anxiolytic and depression-releasing effects by O-1602 treatment, which were revealed after the knockdown of GPR55 in the medial orbital cortex of chronic restrain stress-induced C57BL male mice [80]. Therefore, several in vivo studies support the anti-depressive and anxiolytic effects of GPR55 activation in different mouse models of chronic stress and depression-like behavior. Interestingly, the expression of GPR55 was reduced in the dorsolateral frontal cortices of suicide victims compared to natural deceased persons with increased expression of CB2-GPR55 heterodimers, as mentioned before. However, since the authors excluded suicide victims with a history of depression or proof of antidepressants in their body fluids [43], no conclusions about the role of GPR55 in depression can be drawn from this work.

For schizophrenia, some authors suggest potential antipsychotic effects of CBD via inhibition of GPR55 [81]. Since more than 65 molecular targets of CBD are known [82], these observed effects of CBD might only partially depend on GPR55. However, there is a lack of research regarding the possible involvement of GPR55 in the development and progress of schizophrenia. Schizophrenia is associated with inflammatory processes and altered microglial activation [83]; therefore, activation of GPR55 and modulation of inflammatory processes via this receptor might be beneficial in treating schizophrenia. More research is necessary to understand the role of GPR55 in this disorder.

In the β-amyloid-induced AD mice model, activation of GPR55 using the commercial agonist O-1602 ameliorated the induced cognitive impairment in behavioral tests by suppressing the RhoA/ROCK2 pathway and decreasing β-amyloid levels in the hippocampus and frontal cortex. Interestingly, β-amyloid treatment of the mice reduced GPR55 levels in the hippocampi and frontal cortices, but intraventricular administration of O-1602 normalized GPR55 protein levels in those CNS areas comparable to baseline levels. Decreased levels of IL-1β and TNFα, activation of anti-oxidative mechanisms, and reduction of neuronal apoptosis were observed in the CNS of O-1602-treated animals [84]. Similar effects were described for the streptozotocin-induced murine AD model [85] and LPS-treated mice [86]. In homozygous 5xFAD mice, a genetic model of AD, CB1 receptor levels were reduced while CB2 and GPR55 levels were increased compared to non-transgenic wild-type mice. A positive correlation was found between CB2 and GPR55 levels and β-amyloid burden in the hippocampi. Moreover, the expression of GPR55 showed a negative correlation with Iba-1 and COX-2 synthesis, suggesting an additional anti-inflammatory and anti-oxidative role of the receptor [87]. Confirming the previously discussed anxiolytic effects of O-1602, GPR55 synthesis was negatively correlated with anxiety-like behavior and novel object exploration but positively correlated with locomotion [87].

In CB1 and GPR55 transfected SH-SY5Y cells, both receptors were found on outer cell membranes and mitochondrial membranes, and CB1-GPR55 heterodimers were observed in the transfected cells [88]. GPR55-transfected cells showed greater prevention of 2 mM MPP+ (toxic metabolite of MPTP) induced cell death than CB1 transfected cells and wild-type SH-SY5Y cells. Heterodimerization of CB1 and GPR55 prevented cell death induced by 1 mM MPP+ treatment, and either CB1 or GPR55 only transfected cells. Interestingly, the GPR55 agonist CID1792197 did not prevent MPP+-induced cell death in GPR55 transfected cells but even reduced cell growth in SH-SY5Y cells while forming CB1-GPR55 heterodimers [88]. These results (Table 1) suggest that not only the expression, synthesis, and signaling of GPR55 might be of interest, but functional changes due to heterodimerization may occur and should be part of future research.

**Table 1:** Pro- and anti-inflammatory effects of different GPR55 ligands.

Compound	Observed effects			
Agonists	Upregulated	Unchanged	Downregulated	Study
O-1602	Neurogenesis, neuronal formation	microglial activation		[68]
	IL-4, IL-10		Inflammasome activation, IL-1β, IL-6, TNFα	[70]
			Anxiety-like behavior	[42, 70]
			Depression-like behavior	[41, 70]
		PGE_2_		[71]
	hippocampal & frontal GPR55 expression		RhoA/ROCK2, IL-1β, TNFα, neuronal apoptosis, cognitive impairment	[84]
			IL-1β, IL-6, TNFα, Acetylcholinesterase (AChE) activity, microglial activation	[85]
			NF-κB signaling, IL-1β, IL-6, TNFα, GPR55 downregulation, neuronal apoptosis, cognitive impairment	[86]
LPI		IL-1β, IL-2, IL-10, IL-12, IFNγ	IL-6, TNFα	[69]
CID1792197	Cell growth	Cell growth in CB1-GPR55 heterodimer cells		[88]
Antagonists				
ML 193	Anxiety-like behavior			[42]
			PGE_2_	[71]
CID16020046			IL-6, COX-2	[72]
Coumarin derivates			PGE_2_, COX-2, (IL-6)	[60, 71, 79]
CBD	Antipsychotic effects			[81]

Overall, those studies suggest that GPR55 is of interest in psychiatric and neuropsychiatric disorders. Associations of GPR55 synthesis, signaling, and actions in anti-neuroinflammatory processes and behavioral reactions have been described. However, agonists and antagonists show contradicting results in some of these studies, with not only GPR55 agonistic activation leading to anti-inflammatory processes. For this reason, future research should keep the effects of receptor heterodimerization, functional selectivity, antagonists with inverse agonistic activity, and biased agonism in mind, which may explain the differing findings.

### Modulation of oxidative stress via GPR55

Besides neuroinflammation, oxidative stress harms cells if the anti-oxidative mechanisms of the cells are exhausted, contributing to neurodegenerative diseases such as AD [89]. ROS are defined as oxygen radicals, such as the superoxide radical, hydroxyl radical, and hydrogen peroxide [89], leading to other peroxidized molecules of the cells, such as lipids. The NADPH oxidase (NOX) and the nitric oxide synthase (NOS) families are key sources of intracellular ROS production as side products of the catalyzed reaction [90]. ROS-generating enzymes are found in all cells of the CNS [91], with the location of their majority in the mitochondrial and endoplasmic reticulum (ER) [89, 92]. While high levels of ROS cause neurodegeneration [89], lower levels of ROS fulfill an important role as signal molecules in cellular pathways. As anti-oxidative opponents, anti-oxidative vitamins, such as vitamin C or E, as well as ROS-neutralizing enzymes, such as superoxide dismutases (SOD), glutamate-cysteine ligase (GCL), and glutathione with glutathione S-transferase (GST), are major oxidative cellular defense mechanisms [93]. A key transcription factor initiating and enhancing anti-oxidative cellular responses is nuclear factor-erythroid factor 2-related factor 2 (Nrf2), activated by the phosphatidylinositol 3-kinase (PI3K)/Akt-pathway, amongst others [93]. ROS can furthermore activate MAPK pathways, such as JNK [94], Erk1/2 [95], and NF-κB signaling [96], and therefore regulate autophagy and apoptosis of cells.

Only a few studies focus on GPR55 in oxidative stress. Especially effects of NOS are investigated in these studies. It has been shown that iNOS synthesis was negatively correlated with the GPR55 protein levels in 5xFAD mice [87]. In primary rat microglia, the GPR55 antagonist CID-16020046 reduced LPS-induced NO production and NOS2 expression. However, the GPR55 and CB1/CB2 agonist anandamide and the CB1 antagonist and GPR55 agonist AM-251 also reduced NOS2 expression, while only anandamide reduced NO release. However, the effects of anandamide seem to be mainly dependent on CB2 activation [72]. In mice undergoing chronic social defeat stress, increased NOS2 expression was observed, ameliorated by intraperitoneal O-1602 treatment [70].

Investigations of GPR55-mediated cell death after treatment of different cancer cell lines with N-docosahexaenoyl dopamine (DHA-DA), considered as biased agonist of GPR55, showed increased expression and synthesis of nNOS as well as increased production of NO after DHA-DA stimulation. At the same time, iNOS and eNOS were not affected [97]. Furthermore, DHA-DA induced GPR55- and NOS-dependent ROS generation, partially dependent on NO levels. The PLC/CREB pathway with intracellular calcium modulation was identified as a responsible pathway for the observed nNOS and ROS induction by DHA-DA [97]. In BV-2 cells, LPI inhibited LPS-induced iNOS synthesis and NO production, and reduced NO production was observed after treatment with O-1602 in those cells as well. ROS production, measured with the DCFH-DA assay, was inhibited by LPI in LPS-stimulated BV-2 and primary microglial cells [69]. Overall, agonists, as well as antagonists with possible inverse agonistic activity, reduce ROS and RNS via GPR55 supporting the anti-oxidative properties of this receptor.

Coumarin-derived antagonists with an inverse agonistic activity at GPR55 inhibited 8-iso-PGF_2α_ production in IL-1β-stimulated SK-N-SH cells and LPS-stimulated primary mouse microglia. In addition, GPR55 knockout abolished the prevention of peroxide-induced cell death in SK-N-SH cells suggesting a GPR55-dependent anti-oxidative mechanism [58].

The outstanding role of Nrf2 in the cellular response to oxidative stress and inflammation has been mentioned before. Nrf2 is phosphorylated and activated via PKC, PI3K, MAPK, and Erk1/2 [98], signaling molecules associated with GPR55 [50, 99]. Furthermore, activation of GPR55 using O-1602 increased levels of Nrf2-regulated anti-oxidative enzymes, such as SOD, GSH, and catalase, while it decreased levels of malondialdehyde, another lipid peroxidation marker, in β-amyloid [84] and streptozotocin [85] induced AD mice model. In human ATDC5 cells, stimulation with AGEs reduced Nrf2 synthesis, while treatment with the GPR55 antagonist CID16020046 increased levels of Nrf2, again correlating with the reduction of ROS [100]. We showed an increased expression of Nrf2 in IL-1β stimulated SK-N-SH cells compared to untreated cells, which was significantly and further enhanced by pretreatment of the cells with the highest concentration of the coumarin derivates [58]. Table 2 summarizes the pro- and anti-oxidative effects of different GPR55 ligands.

**Table 2:** Pro- and anti-oxidative effects of different GPR55 ligands.

Compound	Observed effects			
Agonists	Upregulated	Unchanged	Downregulated	Study
Anandamide			NOS2 expression, NO production	[72]
AM251			NOS2 expression	[72]
O-1602			NO production	[69, 70]
	SOD, GSH, Catalase		Malondialdehyde	[84, 85]
	SOD activity		Malondialdehyde	[86]
LPI			iNOS synthesis, NO production, ROS production	[69]
			ROS production	[116]
DHA-DA	nNOS expression, NO synthesis, ROS production	eNOS, iNOS		[97]
Antagonists				
CID-16020046			NOS2 expression, NO production	[72]
			NOX4 expression & synthesis, ROS production	[117]
	Nrf2		ROS production	[100]
KIT C, KIT H	Nrf2		8-iso-PGF_2α_ production, oxidative cell death	[58]

In contrast to neuroinflammation, no studies specifically focus on the role of GPR55 in oxidative stress as a co-cause of psychiatric disorders. The studies presented here suggest a modulatory role of GPR55 in the regulation of oxidative homeostasis; however, anti-oxidative effects have been shown for GPR55 agonists and antagonists. It is important to notice, that the investigated antagonists might enfold their anti-oxidative properties via inverse agonistic activity at GPR55. Therefore, GPR55 is a promising target in psychiatric and neuropsychiatric disorders associated with inflammatory and oxidative dysregulation [27, 33]. Further research is necessary to fully understand the underlying pathways and effects of GPR55 ligands on the Nrf2/Keap1/ARE pathway and the resulting modulation of ROS and RNS.

### New pharmacological approaches in treating psychiatric disorders focusing GPR55

Pharmacological therapeutics available in the treatment of psychiatric disorders are mainly targeting the different neurotransmitter systems so far. The antidepressants venlafaxine, amitriptyline, and clomipramine show the highest effect sizes in the monotherapeutic treatment of depression in multiple studies combined in a meta-analysis of Stone et al. [101]. However, remission rates in the treatment of major depression were only between 31–37% for SSRIs and 40–55% for the SNRI venlafaxin after 8 weeks treatment in a meta-analysis including 8 clinical trials and a total of 2045 patients [102]. In first episode psychosis, only 39.59% of patients showed a decrease of positive symptoms after 8 weeks of treatment with either olanzapine or risperidone, reaching a cumulative response rate of 65.19% after 16 weeks of treatment [103]. Nearly a third of the patients did not show positive response after 16 weeks treatment, and the long treatment periods raise the question if the improvement of symptoms is due to the natural course of the psychosis or really related to effects of the drugs used. Therefore, predictors for treatment response for the well-known antidepressants and antipsychotics in combination with alternative drugs for the treatment of psychiatric disorders are necessary. Focusing on inflammatory processes and oxidative stress as outlined below might be beneficial for the non-responders to the known/currently used therapies.

Neuroinflammation and oxidative stress are closely associated to and promote each other. It has been shown, that major enzymes of intracellular ROS generation such as NOX2 [90] or the inducible NOS [104] are upregulated and activated by inflammatory stimuli. Toll-like receptors (TLR) can foster ROS production NOX-dependent and by enhancing the mitochondrial ROS synthesis [105]. On the other hand, increased levels of ROS enhanced IL-6 production in JEG-3 trophoblast cells via Erk1/2 activation with a significant inhibition of IL-6 synthesis when treating the cells with a ROS scavenger [106]. Oxidized proteins and lipids are recognized as DAMPs and can activate inflammasomes via TLRs leading to release of cytokines and the inflammatory response [105]. Nrf2 deficient mice furthermore showed an increased susceptibility for inflammation, proving anti-inflammatory, besides anti-oxidative, properties of the Nrf2 pathway [107]. Therefore, low levels of ROS in the cells and a basal activation of the Nrf2 pathway equip the cells with a basic inventory of anti-oxidative and anti-inflammatory enzymes as well as pathway activation. GPR55 has the potential to interfere with inflammatory processes and oxidative stress and has been shown to be possibly involved in the etiology of psychiatric and neuropsychiatric disorders. Therefore, ligands of GPR55 might be beneficial for future pharmacological treatment of those disorders.

Besides the synthetic GPR55 agonists and antagonists, GPR55 selective coumarin derivates acting as antagonists with inverse agonistic activity showed anti-inflammatory and anti-oxidative effects in cell culture experiments [58, 60, 71, 79]. Coumarins are already used in medical context [108]. Besides the already mentioned effects of coumarins, derivatives with anti-proliferative, anti-microbial, anti-psychotic, and anticoagulative properties are known [109]. Especially anticoagulative effects need to be ruled out in the development of coumarins for treatment in inflammatory pathologies, to avoid bleeding complications. Warfarin, based on the coumarin scaffold, is a commonly used anticoagulant acting via Vitamin K antagonism and is associated with only few side effects, with hemorrhages being the most severe one [110]. Therefore, other coumarin derivates might be associated with even less adverse effects. Depending on the modifications of the coumarin scaffold, the derivates can show anti-proliferative, anti-oxidative, and anti-inflammatory effects, as well as inhibition of monoamine oxidases (MAO), key enzymes in neurotransmitter degradation, and possible other biological activities in the CNS [111].

**Fig. 2:**
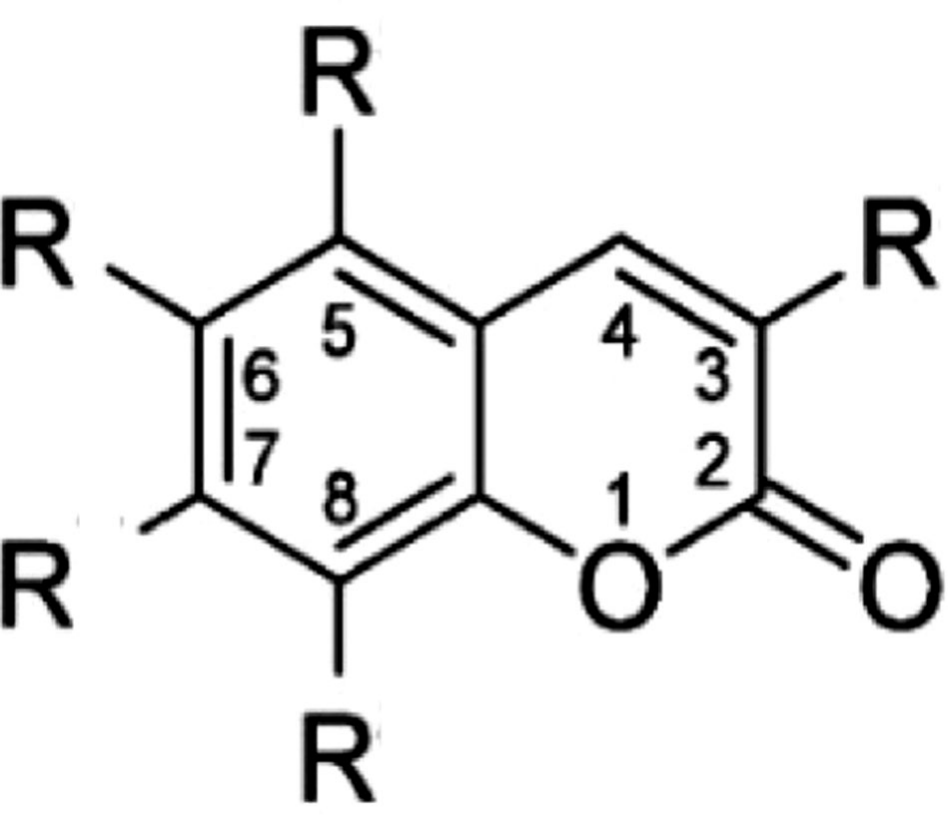
Coumarin scaffold with possible residual groups.

In 3-methyl- and 3-benzyl substituted coumarins, long lipophilic groups on C7 (Fig. 2) led to selectivity for CB-receptors [112]. Short or no substituents on C7 combined with an alkyl rest on C8 showed high GPR55 antagonistic affinity, when C5 was substituted with any rest [113]. Our results show, that those GPR55-selective coumarin derivates enfold inverse agonistic activities in biological cell experiments and are associated with anti-inflammatory and anti-oxidative effects [58, 60, 71, 79]. Biased and inverse agonism opens new options as well as challenges for drug design, since not all agonists or antagonists will lead to the same pattern of receptor-associated pathway activation, dependent of cell type, receptor density, and other factors. Therefore, the effects observed in cell culture should be verified in animal disease models in respect to therapeutical activity phenotypes [76].

Polypharmacy implies a pharmakon acting on multiple molecular targets, and is therefore source of adverse effects and drug interaction on the one hand as well as great therapeutical effectiveness on the other hand depending on the targets affected [114]. Due to the wide range of possible biological effects associated with coumarin scaffolds [111], coumarin derivates are promising structures for intentional polypharmaceutical drug design approaches. In a recently published study using crystal structure complex X-ray, two coumarin derivates showed affinity to both monoaminoxidase (MAO) B and AChE, two enzymes of great therapeutical interest in the treatment of AD. The chemical modifications to the coumarin scaffold were a long lipophilic C7 substitute and either two methyl groups at C3 and C4 or a hydroxymethyl group at C4 [115]. Even if these two compounds would not show affinity to CB-receptors due to their C7 lipophilic rest [113], the study underlines the potential of the coumarin scaffold in the treatment of psychiatric and neuropsychiatric disorders. Other studies confirm the affinity of coumarin-dithiocarbamate to MAO B and AChE, with one of the derivates even reversing scopolamine-induced memory deficits in mice suggesting a benefit in the treatment of AD [114]. While multi-target drug design based on coumarin scaffold focus on AChE and MAO B dual inhibition so far, there might be a benefit to develop coumarin derivates targeting MAO and GPR55. These compounds would on the one hand use an already well-established molecular mechanism in the treatment of depression since MAO inhibition is the underlying mechanism of the antidepressants moclobemide and tranylcypromine. On the other hand, such compounds could include anti-inflammatory and anti-oxidative properties and thus countering for the inflammatory hypothesis of psychiatric disorders. Therefore, further research towards GPR55-selective coumarin derivates with additional affinity for MAO or AChE might open new therapeutical approaches to psychiatric and neuropsychiatric disorders, accounting for different proposed molecular mechanisms of these disorders.

## Concluding remarks and future perspectives

Neuroinflammatory processes and oxidative stress have gained increasing interest in research of the molecular pathogenesis of psychiatric and neurological disorders. In some patients suffering from those disorders, increased inflammatory parameters were observed and an oxidative dysregulation is suggested by different studies. First anti-inflammatory approaches to treat major depression reveal promising results regarding the reduction of depressive symptoms. Furthermore, the symptoms and progress of AD are positively affected by anti-inflammatory treatment in several studies. Since GPCRs are forming the largest receptor family in vertebrates, they are already the main target of numerous drugs. The recently deorphanized GPR55 receptor is discussed as part of the endocannabinoid system and studies demonstrate its role especially in inflammation as well as psychiatric and neurological disorders. However, most of the studies are conducted in cell cultures and animal models. GPR55 ligands show promising effects on inflammatory processes and oxidative stress in those investigations. On the other hand, anti-inflammatory treatment may be associated with severe side effects depending on the drugs used. Even if there is need of further studies in animal disease models and research to elucidate the role of GPR55 in different psychiatric and neuropsychiatric disorders in humans, targeting GPR55 might open a new pharmacological approach for the treatment of inflammation and oxidative stress as possible molecular pathomechanism of these disorders. Coumarin derivates are used in other medical fields already and the best known coumarin-based drug is probably warfarin, which has been used as Vitamin K antagonist in humans for ages now. However, chemical modifications of the coumarin scaffold are determining the selectivity for different targets and greatly affect the size of the observed effects. Therefore, coumarin derivates selective for GPR55 might be interesting candidates for further investigations in the treatment of psychiatric and neuropsychiatric disorders, especially when including MAO or AChE affinity. Future research should focus on the effects of coumarin derivates in different animal disease models for those disorders and assess possible side effects. Pharmacologically, advanced drug design techniques might be used to design multi-target compounds that interfere with different receptors and effectors in inflammatory and oxidative cellular regulation cascades for increasing the effectiveness of the treatment of psychiatric and neuropsychiatric disorders.
